# Muscle activity and acute stress in fibromyalgia

**DOI:** 10.1186/s12891-021-04013-1

**Published:** 2021-02-14

**Authors:** Teemu Zetterman, Ritva Markkula, Juhani V. Partanen, Teemu Miettinen, Ann-Mari Estlander, Eija Kalso

**Affiliations:** 1 Pain Clinic, Department of Anaesthesiology, Intensive Care and Pain Medicine, Helsinki University and Helsinki University Hospital, Helsinki, Finland; 2 City of Vantaa Health Centre, Vantaa, Finland; 3 HUS, Imaging Centre, Clinical Neurophysiology, Helsinki University and Helsinki University Hospital, Helsinki, Finland; 4 SLEEPWELL Research Programme, Faculty of Medicine, University of Helsinki, Helsinki, Finland

## Abstract

**Background:**

Fibromyalgia (FM) patients are likely to differ from healthy controls in muscle activity and in reactivity to experimental stress.

**Methods:**

We compared psychophysiological reactivity to cognitive stress between 51 female FM patients aged 18 to 65 years and 31 age- and sex-matched healthy controls. They underwent a 20-minute protocol consisting of three phases of relaxation and two phases of cognitive stress. We recorded surface electromyography normalized to maximum voluntary muscle contraction (%EMG), the percentage of time with no muscle activity (EMG rest time), and subjective pain and stress intensities. We compared group reactivity using linear modelling and adjusted for psychological and life-style factors.

**Results:**

The FM patients had a significantly higher mean %EMG (2.2 % vs. 1.0 %, *p* < 0.001), pain intensity (3.6 vs. 0.2, *p* < 0.001), and perceived stress (3.5 vs. 1.4, *p* < 0.001) and lower mean EMG rest time (26.7 % vs. 47.2 %, *p* < 0.001). In the FM patients, compared with controls, the pain intensity increased more during the second stress phase (0.71, *p* = 0.028), and the %EMG decreased more during the final relaxation phase (-0.29, *p* = 0.036). Within the FM patients, higher BMI predicted higher %EMG but lower stress. Leisure time physical activity predicted lower %EMG and stress and higher EMG rest time. Higher perceived stress predicted lower EMG rest time, and higher trait anxiety predicted higher pain and stress overall.

**Conclusions:**

Our results suggest that repeated cognitive stress increases pain intensity in FM patients. FM patients also had higher resting muscle activity, but their muscle activity did not increase with pain. Management of stress and anxiety might help control FM flare-ups.

**Trial registration:**

Retrospectively registered on ClinicalTrials.gov (NCT03300635).

## Background

Fibromyalgia (FM) is currently considered to be a nociplastic pain condition, and several studies have reported plastic changes in the central nervous system (CNS) [1, 2]. However, peripheral mechanisms may also play a role in the development of FM.

The CNS changes seen in functional magnetic resonance imaging (fMRI) in FM resemble those seen in other chronic pain conditions where peripheral nociplastic pain is evident, such as osteoarthritis (OA) and rheumatoid arthritis (RA). In OA and RA, the pain persists in some patients even after successful joint replacement in OA or normalization of inflammatory biomarkers in RA, indicating a nociplastic mechanism, similar to that in FM, initiated by peripheral pathology [3, 4].

While no evident structural pathology has been found in the muscles of FM patients, the pain pattern of FM patients might be of muscular origin, as it resembles the pain pattern provoked by muscle trigger points in myofascial pain syndrome [5]. The muscle metabolism of FM patients may also be disturbed, as microdialysis studies of muscle interstitium of FM patients have shown heightened glutamate and lactate levels, indicative of increased anaerobic muscle metabolism [6].

Functional changes in muscle activity can be studied using electromyography (EMG). Surface electromyography (sEMG) is a non-invasive and painless version of EMG, allowing undisturbed measurement during both relaxation and activity. It is unable to distinguish individual motor units but instead measures the sum of several muscle motor unit potentials [7].

Electromyography (EMG) studies comparing FM patients and healthy controls have reported both decreased and increased muscle activity. Thieme et al. reported lower sEMG activity of the upper trapezius muscle at baseline and during alternating instructed relaxation and stress (mental arithmetic or social conflict) when comparing 30 FM patients with 30 healthy controls [8]. On the other hand, Westgaard et al., who examined sEMG normalized with respect to maximum voluntary contraction (MCV) (i.e. %EMG), reported increased %EMG in 26 FM patients compared with 25 healthy controls while holding inspiratory breathing and during a mental stress test or instructed relaxation. However, increased %EMG was not observed during unrestrained rest time [9].

Pain-related reactions should not be studied without considering psychosocial factors. A body of evidence confirms the importance of negative (both general and pain-specific) risk factors (anxiety, fear, catastrophizing) and positive, protective, factors (resilience, coping with pain) for pain reactions. Negative factors are generally associated with increased pain and even with the development from acute to chronic pain, while positive, protective, factors associate with less pain [10].

FM patients often report mental stress as one factor leading to worsening of their symptoms [11] and it has been shown to have a long-term effect on FM symptoms [12]. Mental stress increases muscle tone in both FM patients and healthy individuals [13, 14], this stress-induced muscle activity being associated with increased pain, at least in the trapezius, with a greater increase in FM patients than in healthy controls [13].

We hypothesized that FM patients have higher resting muscle activity, and that experimental stress causes a greater increase in muscle activity and pain in FM patients than in healthy controls. We further hypothesized that these differences in reactivity between groups was associated with differences in prior stress levels, both state and trait anxiety, and with pain catastrophizing.

## Methods

### Study population

We recruited 51 Finnish women with FM, aged 18 to 65 years, through the Helsinki University Hospital clinics, City of Vantaa health centre, and the private clinic of one of the authors (RM). Thirty-one healthy, female, age-matched controls were recruited from the staff of these health care units and from a local home economics organisation (Uudenmaan Martat ry). The controls were not required to be completely pain-free and were not excluded for minor, localized pain complaints such as mild hip or knee pain.

Inclusion criteria for the FM patients were the American College of Rheumatology (ACR) classification criteria for FM from 1990 (ACR1990) [15].

Both the patients and controls also participated in an oral glucose tolerance test and in bicycle ergometry, the results of which will be published later. These guided the selection of exclusion criteria, which were conditions likely to affect muscle function and metabolism measurements or to interfere with the ability to participate in the measurements: diabetes, heart disease, peripheral atherosclerotic disease, uncontrolled hypertension, neurological, neuromuscular or muscle disease, severe psychiatric disorders, continuous use of beta-blockers, beta-agonists or statins, any musculoskeletal condition that would prevent bicycle ergometry, and poor Finnish language skills that would affect the ability to answer the questionnaires.

**Fig. 1:**
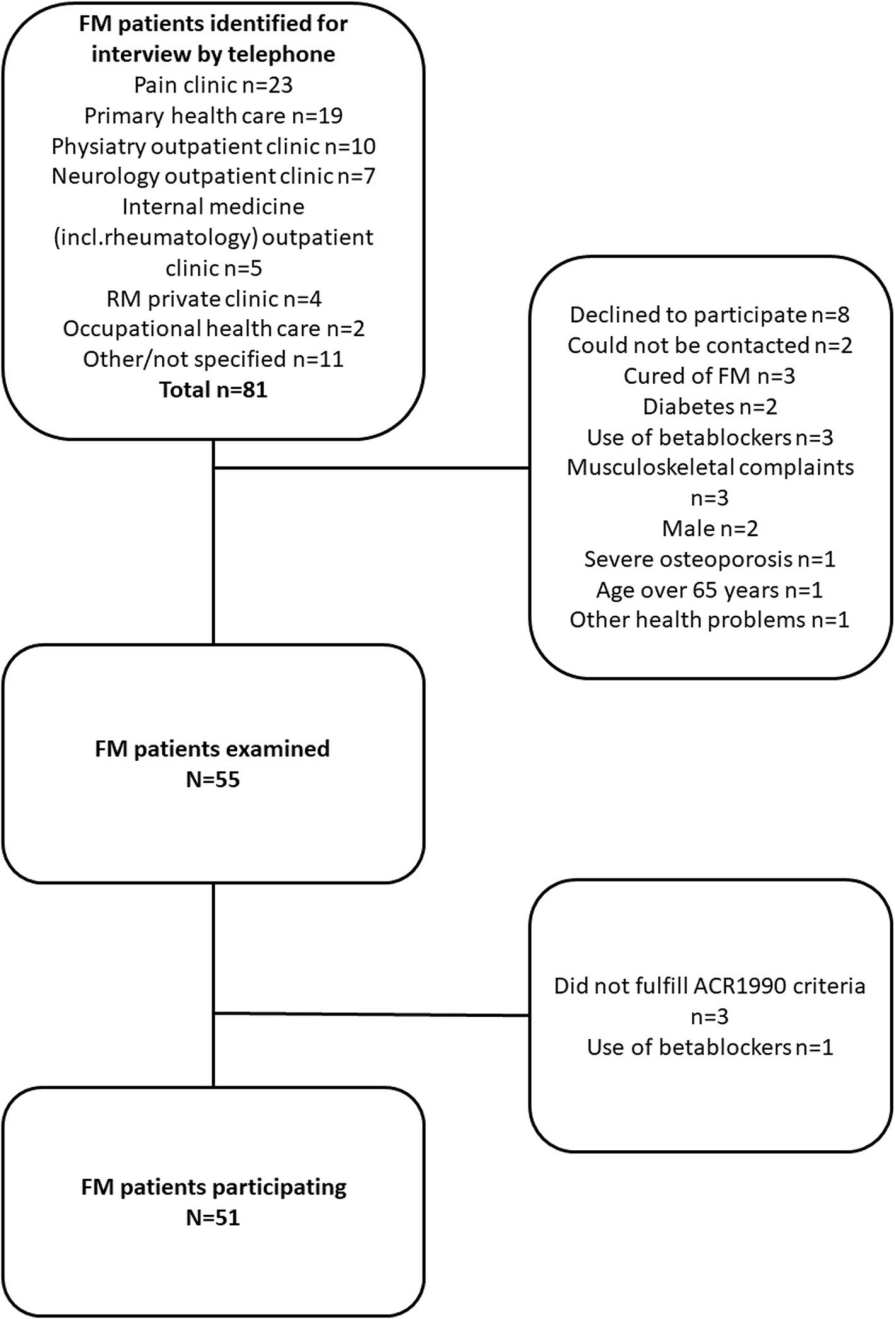
Patient recruitment flowchart

Consecutive patients meeting the ACR1990 criteria according to patient records were invited for a visit where the diagnosis of FM was confirmed by the research physician [15]. Eighty-one patients were invited to participate in the study, 8 declined, and 22 were excluded (Fig. 1), resulting in 51 FM participants.

Thirty-two controls were originally recruited, but one was excluded because of a history of FM-like symptoms and the finding of 12/18 tender points in the physical examination, despite their reporting no current symptoms or spontaneous pain.

We collected the data between November 2015 and June 2018.

### Background data

Subjects answered a questionnaire on medical history and provided details on height, weight, pain history, sleep problems, leisure time physical activity habits, working status, education, smoking, handedness, and any former participation in relaxation training (Table 1).

**Table 1:** Baseline demographics (Background data)

	FM (*n* = 51)	Control (*n* = 31)	*p*-value
**Age (years)**			
Mean (SD)	45.1 (12.7)	46.0 (11.7)	0.785
Median [Min, Max]	47.0 [18.0, 66.0]	49.0 [22.0, 61.0]	
**BMI**			
Mean (SD)	28.2 (5.87)	24.7 (3.27)	0.008
Median [Min, Max]	27.4 [18.6, 45.4]	24.5 [19.1, 32.2]	
Missing	3 (5.9 %)	2 (6.5 %)	
**Smoking**			
Yes	12 (23.5 %)	2 (6.5 %)	0.091
No	39 (76.5 %)	29 (93.5 %)	
**Other medical diagnoses**			
Mean (SD)	1.98 (1.75)	0.452 (0.675)	< 0.001
Median [Min, Max]	2.00 [0, 7.00]	0 [0, 2.00]	
**Any daily systemic medication**			
Yes	40 (78.4 %)	7 (22.6 %)	< 0.001
No	11 (21.6 %)	24 (77.4 %)	
**Leisure time activity**			
Inactive	31 (60.8 %)	15 (48.4 %)	0.386
Active	20 (39.2 %)	16 (51.6 %)	
**Mean pain intensity previous week (NRS 0–10)**			
Mean (SD)	5.29 (1.89)	1.65 (1.96)	< 0.001
Median [Min, Max]	6.00 [1.00, 9.00]	1.00 [0, 8.00]	
**Highest pain intensity previous week (NRS 0–10)**			
Mean (SD)	7.34 (1.59)	3.03 (2.23)	< 0.001
Median [Min, Max]	7.75 [3.00, 10.0]	3.00 [0, 8.00]	
Missing	1 (2.0 %)	0 (0 %)	
**Lowest pain intensity previous week (NRS 0–10)**			
Mean (SD)	2.91 (1.87)	0.903 (1.62)	< 0.001
Median [Min, Max]	3.00 [0, 7.00]	0 [0, 6.00]	
Missing	1 (2.0 %)	0 (0 %)	
**Sleep problems**			
Yes	40 (78.4 %)	3 (9.7 %)	< 0.001
No	11 (21.6 %)	28 (90.3 %)	

*FM* Fibromyalgia patients, *BMI* Body Mass Index, *p*-value: Mann-Whitney U test for continuous variables or Chi-squared test for categorical variables, *SD* standard deviation

We calculated subjects’ body mass index (BMI) from their height and weight.

Leisure time physical activity frequency was rated on a four-point scale of *“none”*, *“1–2 times per month”*, *“1–2 times per week”*, or *“more often.”* Exercise intensity was rated on a four-point scale of *“walking”*, *“intermittent walking and running”*, *“slow running or jogging”*, or *“fast running”*. These we dichotomized into a single variable, with subjects classified as physically active if they reported both the highest physical activity frequency (which we took to represent frequent exercise) and physical activity intensity above the lowest level of *“walking”* (which we took to reflect physical activity more akin to sports and not merely everyday activity). These cut-offs were further verified as producing the most closely balanced distributions in the FM patient and control groups.

#### Psychological questionnaires

We expected pre-existing stress, concurrent anxiety, and predisposition to anxiety to influence reactivity to experimentally induced stress and we therefore included the following validated questionnaires.

Perceived Stress Scale (PSS) measures prolonged subjective stress over the previous month: we used the revised 10-item version of this which yields scores from 0 to 40 (higher scores indicate more stress and less feeling of control). The individual items are rated on a five-point scale from 0 (*“Never”)* to 4 (*“Very often”*). For example, Item 6 asks “*In the last month, how often have you found that you could not cope with all the things that you had to do?*” [16, 17].

The State-Trait Anxiety Inventory (STAI) [18] consists of two parts: STAI-A measures the level of anxiety at the time of testing (state anxiety); and STAI-B measures a persistent tendency towards anxiety (trait anxiety). Both STAI-A and STAI-B comprise 20 items, yielding scores from 20 to 80 (higher scores reflect higher levels of anxiety). Examples from STAI-A include “*I am presently worrying*”, “*I feel nervous*” and, from STAI-B, “*I am inclined to take things hard*”, “*I lack self-confidence*”. The four-point rating scale of items is from 1 (“*Not at all*”) to 4 (“*Very much so*”).

#### Pain‐related questionnaires

The Fibromyalgia Impact Questionnaire (FIQ) [19, 20] assesses the severity of FM and its impact on daily function. It contains 10 items: sub-scores are summed to form a scale of 0 to 100, with a higher score indicating worse symptoms and greater impairment of function. For example, Item 4 asks *“When you worked, how much did pain or other symptoms of fibromyalgia interfere with your ability to work, including housework?”* Questions are answered on a visual analogue scale (VAS) from 0 *(“No problem”*) to 10 (*“Great difficulty”*).

The Pain Catastrophizing Scale (PCS) [21] consists of 13 statements rated on a five-point scale from *“Not at all”* to *“All the time”*. For example, Item 3 states *“The pain is terrible, and I think it is never going to get any better”*. A sum score of 0 to 52 is calculated, with higher scores indicating a greater tendency to catastrophize when in pain.

### Study protocol

The research visit took place at the Helsinki University Hospital pain clinic. The subjects had been instructed to discontinue any beta-agonists, beta-blockers, or muscle relaxants normally used symptomatically before the visit. First, the subjects answered the validated questionnaires and other questionnaires to detail background information, medical history, and symptoms.

The research physician then palpated the 18 tender points of the ACR1990 classification criteria.

**Fig. 2:**
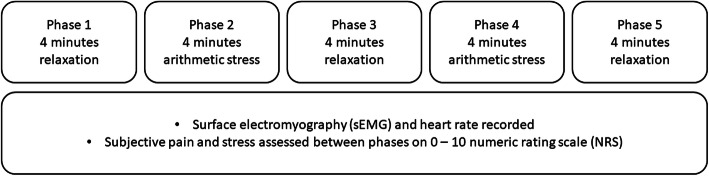
Measurement Protocol

We completed a protocol of alternating phases of relaxation and induced cognitive stress. During this protocol, we recorded sEMG and heart rate, as well as self-reported pain intensity and stress. The results for heart rate measurements will be published separately. The measurement protocol is outlined in Fig. 2.

During the measurement, the subjects were instructed to remain still, seated in an armchair. The measurement consisted of five phases, alternating between relaxation and a mental arithmetic task to induce cognitive stress. Starting with relaxation, there were three relaxation and two stress phases, each lasting four minutes. At the start of the measurement, between each phase and at the end, the subjects were asked to verbally rate both their pain and stress on numerical rating scales (NRS) of 0 to 10 (0 meaning no pain or stress and 10 being the worst imaginable pain or stress).

We used an sEMG monitor (Megawin ME6000 Muscle Tester MT-M6T16-0-10EN, Bittium Corporation, Finland) with a sample rate of 1000 Hz to record sEMGs of left and right upper trapezius, biceps brachii, and erector spinae muscles. The sticky gel electrodes (Ambu Bluesensor M M-00-S/50, Malaysia) for the sEMG measurement were attached as follows.

For the upper part of the trapezius, the anode and cathode electrodes were placed 1 cm medially and laterally from the point in the middle on the line from the posterolateral edge of the acromion to the processus spinosus of the 7th cervical vertebra respectively. The grounding electrode was placed 2.5 cm caudally on the line orthogonal to the acromio-cervical line.

For the erector spinae, the anode was placed 3 cm laterally from the processus spinosus of the 2nd lumbar vertebra and the cathode 2 cm caudally from the anode. The grounding electrode was placed 2 cm laterally from the midpoint of the line between the anode and the cathode.

For the biceps brachii, the anode and cathode were placed 1 cm cranially and caudally from the point two thirds down on the line from the anterolateral edge of the acromion to the fossa cubitae, respectively. The grounding electrode was placed 2.5 cm laterally from the midpoint of the line between the anode and the cathode.

For relaxation, subjects could choose between silence or calm classical music (excerpts from J S Bach’s Ave Maria, Overture (Suite) No. 3 in D Major, BWV 1068: II. Air (“Air on a G String”), and the Goldberg variations).

For the arithmetical task, subjects listened to a recording of 14 series of 10 random numbers from 0 to 9 with background white noise calibrated to 60 dB. The subjects were instructed to mentally sum the numbers and asked to give their answer at the end of each series. They were told whether their answers were correct or incorrect but for four of the series they were told they were wrong regardless of their answer. We modelled this protocol on work by Thieme et al. [8].

At the end of the procedure, we measured the maximum voluntary contractions (MVCs) of the monitored muscles. For each pair of muscles, the trapezii, the bicipites, and the erectores spinae, subjects were instructed to produce maximum force in an isometric contraction for 10 seconds against resistance provided by the same research physician. The MVC was recorded twice for each pair of muscles at an interval of 1 minute.

The subjectively rated pain levels and perceived stress during the protocol, together with the EMG recordings, formed the psychophysiological measures evaluating our main outcomes: pain and muscle activity.

### Signal analysis

We used Matlab R2017b for signal processing. The raw sEMG was first detrended to a mean amplitude of zero. The signal’s power spectrum was then inspected visually to identify sharp peaks of alternating current (AC) noise caused by various electronic devices, most commonly at 50 Hz and multiples thereof. Noise peaks were removed using interpolation to flatten the power of the noisy frequency spectrum range of 1 Hz to the mean power of frequency range 0.5 Hz above and below the noisy range. Finally, a fifth-order Butterworth low-pass filter was applied to remove frequencies above 450 Hz not containing significant EMG activity.

### EMG variable calculation

We chose %EMG and EMG rest time as the outcome measures of muscle activity. As these are calculated using the MVC, they would likely minimize individual differences in, for example, muscle mass and thickness of subcutaneous tissue, which might affect absolute sEMG values.

We calculated the root mean square of EMG amplitude with a 100-ms non-overlapping window for the relaxation and mental arithmetic phases. For the MVC recording, a 1000-ms non-overlapping window was used for better reproducibility in obtaining MVC [22]. We used the median of the highest five 1000-ms samples as the MVC.

We then calculated the normalized EMG in percentage (%EMG) by dividing the absolute EMG of each muscle by the muscle MVC. This allowed the use of mean %EMG of all recorded muscles to evaluate overall muscle tension.

EMG rest time is the proportion of time the muscle is at rest with no significant muscle activity. We used the definition of sEMG less than 0.5 % of MVC and the 100-ms window described above to calculate this [9].

For the baseline values of %EMG and EMG rest time, we used the mean over the first 30 seconds at the beginning of the recording.

### Statistical analyses

For statistical analyses, we used R version 3.5.3 (The R Foundation for Statistical Computing 2019). For general linear modelling, we used the lme4 package for R [23].

A combination of visual inspection of histograms and the Shapiro-Wilk test determined that most demographic, psychological, and symptom scores were non-normally distributed, due to the low scores reported by the controls. Therefore, we compared FM patients and controls using the Mann-Whitney U test for continuous variables, and the chi-squared test for categorical variables.

We calculated Pearson correlation coefficients to test for correlation between questionnaire data and %EMG, and pain and stress intensities during the measurement. We tested for correlations in the whole study population and within FM patient and control groups.

The main objective of our study was to determine whether FM patients and controls reacted differently to the repeated alternating relaxation and stress. We tested the change from baseline for %EMG, EMG rest time, pain, and stress during the different phases with general linear models. The base model tested the interaction between group and time for each outcome Y, written as *Y ~ Group + Phase + Group:Phase*. A second model was adjusted for BMI, smoking (smoker vs. non-smoker), leisure time physical activity (active vs. inactive), trait anxiety (STAI-B score), and preceding stress (PSS score).

We used trait anxiety instead of state anxiety, as the more stable characteristics of the subjects were of greater interest. In any case, the correlation between the sum scores of the two anxiety scales was very high.

Secondarily, we evaluated the effect of lifestyle and psychological factors on stress reactivity within the FM patient subgroup. For this, we tested for interactions between time and the following possible predictors of the outcome measures: trait anxiety, preceding prolonged stress (PSS score), FM symptom severity (FIQ score), catastrophizing (PCS score), BMI, smoking, and leisure time physical activity.

We constructed a reduced model which aimed to find the minimum number of variables which predicted the observed data well. The significance of these likely predictors and interaction terms was tested hierarchically. For each outcome variable, a full model containing all likely predictors was compared to a reduced model with one predictor variable removed. We used maximum likelihood (ML) analysis of variance (ANOVA) testing. If the model with a variable removed did not differ significantly from the full model (*p* > 0.05), the variable was removed as inconsequential to the model’s fit. If the models differed significantly (*p* < 0.05) and the fit of the reduced model was worse (Akaike information criterion increased), the variable was retained. After testing each variable individually, a level-one reduced model, which contained only the retained variables, was constructed.

The process was then repeated, testing (as above) whether the reintroduction of any individually removed variable would significantly improve the model’s fit. The resulting level-two reduced model was then again tested with individual removal of the remaining variables and so on until the fit of the model no longer improved significantly: this was used as the final reduced model.

## Results

### Demographics and questionnaires

Forty-seven of the 51 patients (92 %) fulfilled the ACR 2016 diagnostic criteria for FM in addition to the ACR 1990 criteria. Patients were often unable to report the exact duration of FM symptoms and provided only estimates such as “at least ten years”. The duration of FM was thus stratified into two classes, under 3 years or 3 years and over [24]. Most patients (86 %) had suffered from FM symptoms for at least 3 years.

The patients had higher BMIs and were more likely to smoke or have other chronic diseases than the healthy controls. FM patients also reported more problems with sleeping, lower leisure time physical activity, and greater pain intensity over the previous week than controls, and were more likely to use medication (Table 1).

On the FIQ, 13 patients left at least one item blank. These missing data were imputed by multiplying the score by a factor of 10/n (where *n* = the number of answered questions) [19].

The FM patients perceived their overall stress level (PSS) as higher and had greater state and trait anxiety (STAI-A and STAI-B) than controls. The results, along with FIQ and PCS scores for the FM patients, are shown in Table 2.

**Table 2:** Preliminary questionnaire data (Psychological questionnaires)

	FM (*n* = 51)	Control (*n* = 31)	*p*-value
**ACR2016 symptom severity scale**			
Mean (SD)	8.12 (2.44)	2.35 (1.56)	< 0.001
Median [Min, Max]	8.00 [4.00, 12.0]	3.00 [0, 6.00]	
**ACR2016 widespread pain index**			
Mean (SD)	11.3 (4.19)	1.48 (1.77)	< 0.001
Median [Min, Max]	11.0 [4.00, 19.0]	1.00 [0, 8.00]	
**FIQ score**			
Mean (SD)	44.2 (14.8)	15.3 (5.48)	< 0.001
Median [Min, Max]	47.3 [8.57, 70.1]	13.2 [10.0, 32.3]	
Missing	1 (2.0 %)	0 (0 %)	
**PCS score**			
Mean (SD)	17.6 (10.2)	2.65 (3.56)	< 0.001
Median [Min, Max]	16.0 [0, 48.0]	1.00 [0, 13.0]	
**PSS score**			
Mean (SD)	19.5 (7.74)	7.74 (4.15)	< 0.001
Median [Min, Max]	20.0 [3.00, 35.0]	7.00 [2.00, 19.0]	
Missing	1 (2.0 %)	0 (0 %)	
**STAI-A score**			
Mean (SD)	40.7 (9.85)	29.2 (3.81)	< 0.001
Median [Min, Max]	39.0 [27.0, 73.0]	29.0 [23.0, 38.0]	
**STAI-B score**			
Mean (SD)	45.0 (9.68)	28.3 (5.27)	< 0.001
Median [Min, Max]	46.0 [25.0, 64.0]	27.0 [21.0, 44.0]	
Missing	3 (5.9 %)	1 (3.2 %)	

*FM* Fibromyalgia patients, *p*-value: Mann-Whitney U test, *ACR2016* American College of Rheumatology 2016 modified diagnostic criteria, *FIQ* Fibromyalgia Impact Questionnaire, *PCS* Pain Catastrophizing Scale, *PSS* Perceived Stress Scale, *STAI-A* State-Trait Anxiety Inventory A (State Anxiety), *STAI-B* State-Trait Anxiety Inventory B (Trait Anxiety), *SD* standard deviation

### Psychophysiological measures

The mean absolute sEMGs of the individual muscles of the FM patients was mostly similar to those of the controls. Patients had higher values at 0.05 significance level only in the left trapezius and the right biceps. The MVCs for the upper limbs, trapezii, and bicipites brachii bilaterally were significantly lower for the patients than for the controls, but the MVCs of the trunk (erectores spinae) were similar (Table 3).

**Table 3:** Absolute surface electromyography results in µV during the measurement and the maximum voluntary contractions (Psychophysiological measures)

	FM (*n* = 51)	Control (*n* = 31)	*p*-value
**Trapezius, left**			
Mean (SD)	6.3 (8.5)	3.5 (1.9)	0.027
Median [Min, Max]	3.2 [1.6, 57.1]	2.8 [1.6, 11.1]	
**Trapezius, right**			
Mean (SD)	7.7 (8.2)	5.2 (4.4)	0.082
Median [Min, Max]	3.8 [2.2, 42.5]	3.1 [1.8, 20.7]	
**Erector spinae, left**			
Mean (SD)	2.4 (1.3)	2.1 (1.9)	0.499
Median [Min, Max]	1.9 [1.1, 6.8]	1.7 [1.0, 12.4]	
**Erector spinae, right**			
Mean (SD)	2.4 (1.3)	2.3 (1.0)	0.635
Median [Min, Max]	2.1 [1.1, 9.4]	2.0 [1.0, 5.5]	
**Biceps brachii, left**			
Mean (SD)	2.9 (1.3)	2.6 (1.3)	0.346
Median [Min, Max]	2.6 [1.6, 8.2]	2.4 [1.2, 7.1]	
**Biceps brachii, right**			
Mean (SD)	2.9 (1.8)	2.1 (1.1)	0.014
Median [Min, Max]	2.3 [1.1, 10.7]	1.9 [1.0, 6.9]	
**MVC trapezius, left**			
Mean (SD)	531 (406)	887 (567)	0.004
Median [Min, Max]	433 [82.5, 2510]	766 [133, 2460]	
**MVC trapezius, right**			
Mean (SD)	588 (378)	890 (542)	0.009
Median [Min, Max]	561 [115, 2290]	669 [109, 2080]	
**MVC erector spinae, left**			
Mean (SD)	125 (187)	128 (67.8)	0.926
Median [Min, Max]	84.8 [15.2, 1300]	109 [26.7, 306]	
**MVC erector spinae, right**			
Mean (SD)	109 (87.2)	139 (62.0)	0.0778
Median [Min, Max]	84.0 [11.4, 442]	128 [44.9, 311]	
**MVC biceps brachii, left**			
Mean (SD)	457 (329)	768 (401)	< 0.001
Median [Min, Max]	365 [95.0, 1420]	772 [241, 1830]	
**MVC biceps brachii, right**			
Mean (SD)	442 (335)	720 (430)	0.003
Median [Min, Max]	323 [57.7, 1500]	638 [167, 1910]	

*FM* Fibromyalgia patients, *MVC* Maximum voluntary contraction, *SD* Standard deviation

The mean %EMG of all muscles was significantly higher in the FM patients than in controls (Table 4). The EMG rest time was shorter for the FM patients in the trapezius and biceps muscles than for the controls, but similar in the erector spinae muscles (Table 5).

**Table 4:** Normalized surface electromyography results as percentage (Psychophysiological measures)

	FM (*n* = 51)	Control (*n* = 31)	*p*-value
**Trapezius, left**			
Mean (SD)	1.6 (1.7)	0.6 (0.5)	< 0.001
Median [Min, Max]	1.0 [0.2, 8.0]	0.4 [0.1, 2.0]	
**Trapezius, right**			
Mean (SD)	1.5 (1.2)	0.7 (0.5)	< 0.001
Median [Min, Max]	1.0 [0.2, 5.5]	0.7 [0.1, 2.1]	
**Erector spinae, left**			
Mean (SD)	4.0 (3.3)	2.2 (1.8)	0.002
Median [Min, Max]	3.0 [0.1, 12.8]	1.6 [0.4, 7.9]	
**Erector spinae, right**			
Mean (SD)	4.1 (4.2)	2.0 (1.2)	0.001
Median [Min, Max]	2.4 [0.5, 19.1]	1.7 [0.6, 5.1]	
**Biceps brachii, left**			
Mean (SD)	1.0 (0.8)	0.4 (0.3)	< 0.001
Median [Min, Max]	0.8 [0.2, 3.3]	0.4 [0.1, 1.4]	
**Biceps brachii, right**			
Mean (SD)	1.0 (0.8)	0.4 (0.4)	< 0.001
Median [Min, Max]	0.8 [0.1, 3.9]	0.4 [0.1, 2.0]	

*FM* Fibromyalgia patients, *SD* standard deviation

**Table 5:** Rest time of muscles: percentage of time when surface electromyography voltage < 0.5 % of maximum voluntary contraction (Psychophysiological measures)

	FM (*n* = 51)	Control (*n* = 31)	*p*-value
**Trapezius, left**			
Mean (SD)	35.4 (35.2)	66.4 (31.8)	< 0.001
Median [Min, Max]	23.6 [0.0, 97.5]	80.6 [0.2, 100]	
**Trapezius, right**			
Mean (SD)	35.6 (30.7)	59.6 (30.8)	0.001
Median [Min, Max]	36.6 [0.1, 98.6]	57.4 [0.3, 100]	
**Erector spinae, left**			
Mean (SD)	5.4 (17.2)	7.1 (17.0)	0.658
Median [Min, Max]	0.0 [0.0, 99.9]	0.3 [0.0, 79.2]	
**Erector spinae, right**			
Mean (SD)	4.1 (11.2)	4.3 (8.8)	0.945
Median [Min, Max]	0.1 [0.0, 62.2]	0.6 [0.0, 43.6]	
**Biceps brachii, left**			
Mean (SD)	40.0 (35.4)	71.1 (27.9)	< 0.001
Median [Min, Max]	30.5 [0.0, 99.9]	80.3 [17.6, 100]	
**Biceps brachii, right**			
Mean (SD)	40.0 (36.2)	74.6 (27.5)	< 0.001
Median [Min, Max]	32.4 [0.0, 99.9]	83.6 [11.6, 100]	

*FM *Fibromyalgia patients, *SD *standard deviation

The pain and perceived stress ratings reported by the subjects during the protocol were higher in the FM patients than the controls (Table 6).

**Table 6:** Mean values for main psychophysiological measures (Psychophysiological measures)

	FM (*n* = 51)	Control (*n* = 31)	*p*-value
**%EMG (%)**			
Mean (SD)	2.2 (1.4)	1.0 (0.5)	< 0.001
Median [Min, Max]	1.8 [0.3, 7.3]	0.9 [0.3, 2.6]	
**EMG Rest Time (%)**			
Mean (SD)	26.7 (19.9)	47.2 (16.4)	< 0.001
Median [Min, Max]	25.6 [0.4, 79.5]	49.0 [13.1, 74.3]	
**Pain (NRS 0–10)**			
Mean (SD)	3.6 (2.2)	0.1 (0.5)	< 0.001
Median [Min, Max]	3.7 [0.0, 8.5]	0.0 [0.0, 2.5]	
**Stress (NRS 0–10)**			
Mean (SD)	3.5 (2.4)	1.4 (1.4)	< 0.001
Median [Min, Max]	3.3 [0.0, 9.3]	1.3 [0.0, 6.3]	

*FM *Fibromyalgia patients, *SD *standard deviation, *NRS *Numerical rating scale, *%EMG *Normalized surface electromyography

### Responses to the stress‐relaxation protocol

We used general linear modelling to determine differences in responses to the measurement protocol and likely predictors of outcome measures.

In the base model *Baseline + Group × Time*, significant group–phase interactions, indicating a difference in change from baseline, were found in the second stress phase (phase 4), where FM patients showed a greater pain intensity increase (estimate of interaction 0.71, 95 % confidence interval (CI) 0.04 to 1.38; *p* = 0.038), and in the final relaxation phase (phase 5), where the FM patients had a higher %EMG decrease (estimate of interaction − 0.29, 95 % CI -0.56 to -0.02; *p* = 0.033). No group interaction was found for EMG rest time or stress.

**Fig. 3:**
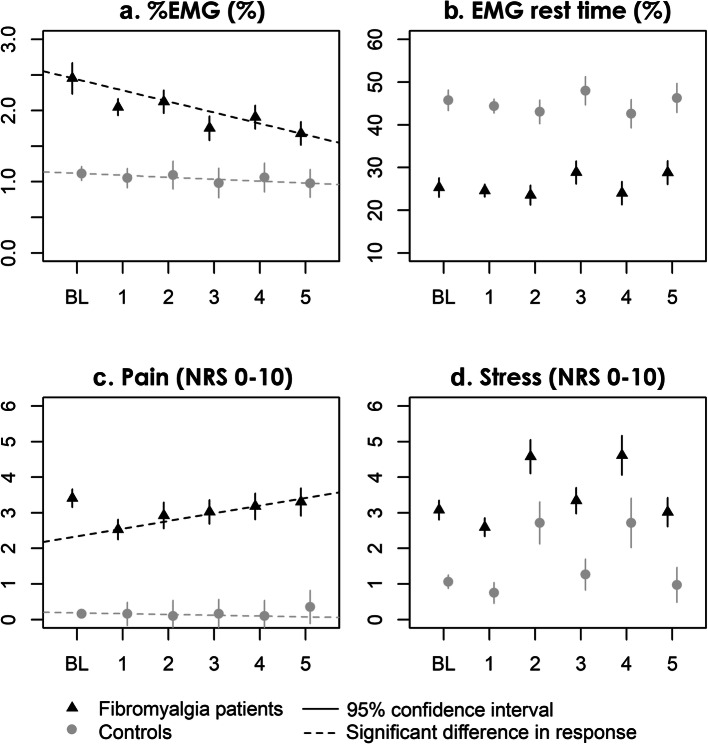
Outcome measures. **a** Surface electromyography normalized to MVC; **b** Surface electromyography rest time (sEMG < 0.5 % of MVC); **c** Subjective pain intensity NRS; **d** Subjective perceived stress NRS. Models are adjusted for BMI, smoking, leisure time physical activity, trait anxiety (STAI-B), and preceding perceived stress (PSS). To illustrate group differences in absolute values, the models are plotted fitted to the mean baseline value of each group

The group-time interactions remained similar after adjusting for BMI, smoking, leisure time physical activity, STAI-B, and PSS, with group (FM) – time (stress phase 2) interaction for pain of 0.71 (95 % CI 0.08 to 1.34; *p* = 0.028), and group (FM) – time (final relaxation phase) interaction for %EMG of -0.29 (95 % CI -0.57 to -0.02; *p* = 0.036) (Fig. 3).

The likely predictors BMI, smoking, leisure time physical activity, STAI-B, PSS, FIQ, PCS, and the baseline value of each outcome measure, were tested for significance and interactions with time within the FM patient subgroup (see 2.6).

The final models were:

$$ {\displaystyle \begin{array}{l}\% EMG\sim Baseline\times Time+ BMI+ Leisure\ time\ physical\ activity;\\ {} EMG\  rest\ time\sim Baseline+ Time+ Leisure\ time\ physical\ activity+ PSS+ FIQ;\\ {} Pain\sim Baseline+ Time+ STAI-B;\\ {} Stress\sim Baseline+ Time+ BMI+ Leisure\ time\ physical\ activity+ STAI-B.\end{array}} $$

The only outcome measure with interactions was %EMG, where a higher baseline %EMG predicted a lower %EMG at each subsequent time point. Higher BMI predicted higher overall %EMG and lower overall stress whereas higher leisure time physical activity predicted lower %EMG and stress and longer EMG rest time overall. Higher PSS predicted shorter EMG rest time, higher FIQ predicted longer EMG rest time, and higher STAI-B predicted higher pain and stress overall. Smoking or PCS did not significantly affect the fit of the models (Table 7).

**Table 7:** Hierarchically reduced generalized linear models for the outcome measures within the fibromyalgia subgroup (Responses to the stress-relaxation protocol)

	%EMG			EMG rest time			Pain			Stress			
*Predictors*	*Estimates*	*CI*	*p*	*Estimates*	*CI*	*p*	*Estimates*	*CI*	*p*	*Estimates*	*CI*	*p*	
(Intercept)	-0.01	-0.37–0.35	0.969	-0.02	-0.04–0.00	0.126	-1.25	-2.06 – -0.44	**0.003**	-0.07	-1.25–1.11	0.906	
baseline	0.83	0.78–0.88	**< 0.001**	0.98	0.95–1.01	**< 0.001**	0.83	0.75–0.90	**< 0.001**	0.86	0.79–0.93	**< 0.001**	
time [2: stress]	0.34	0.04–0.65	**0.029**	-0.01	-0.03–0.01	0.254	0.39	-0.10–0.88	0.117	1.98	1.56–2.40	**< 0.001**	
time [3: relaxation]	0.26	-0.05–0.57	0.102	0.04	0.02–0.07	**0.001**	0.49	0.03–0.95	**0.040**	0.75	0.34–1.15	**< 0.001**	
time [4: stress]	0.40	0.11–0.68	**0.007**	-0.01	-0.03–0.02	0.606	0.65	0.16–1.14	**0.010**	2.02	1.47–2.57	**< 0.001**	
time [5: relaxation]	-0.10	-0.41–0.20	0.520	0.04	0.02–0.07	**0.001**	0.77	0.26–1.28	**0.003**	0.43	-0.05–0.90	0.079	
BMI	0.01	0.00–0.02	**0.034**							-0.03	-0.06 – -0.01	**0.020**	
Leisure time physical activity	-0.15	-0.28 – -0.02	**0.023**	0.02	0.00–0.03	**0.016**				-0.46	-0.78 – -0.14	**0.005**	
PSS				-0.00	-0.00 – -0.00	**0.035**							
FIQ				0.00	0.00–0.00	**0.001**							
STAI-B							0.04	0.02–0.05	**< 0.001**	0.02	0.01–0.04	**0.008**	
time [2] * baseline	-0.11	-0.21 – -0.01	**0.030**										
time [3] * baseline	-0.23	-0.33 – -0.13	**< 0.001**										
time [4] * baseline	-0.22	-0.31 – -0.13	**< 0.001**										
time [5] * baseline	-0.11	-0.21 – -0.01	**0.029**										
Observations	255			255			254			254			

*%EMG* Normalized surface electromyography, *EMGrest* EMG rest time, *BMI* Body Mass Index, *PSS* Perceived Stress Scale, *FIQ* Fibromyalgia Impact Questionnaire score, *STAI-B* State-Trait Anxiety Index Trait score

### Relations between the psychological and psychophysiological measures

Table 8 shows the correlations for the patient group and Table 9 for the control group. The intercorrelations between the psychological questionnaires (STAI-A, STAI-B, PSS) were significant in both patient and control groups. Correlations between psychological questionnaires and pain and stress during the procedure were statistically significant in the patient group, but not in the control group.

**Table 8:** Correlations of the scores in psychological questionnaires with psychophysiological measures within the fibromyalgia patient group (Relations between the psychological and psychophysiological measures)

	FIQ	PCS	STAI-A	STAI-B	PSS	Pain	Stress	%EMG	EMG Rest
PCS	0.40**								
STAI-A	0.56**	0.34*							
STAI-B	0.54**	0.30*	0.75**						
PSS	0.70**	0.29*	0.76**	0.80**					
Pain	0.49**	0.51**	0.38**	0.30*	0.45**				
Stress	0.38*	0.27	0.57**	0.51**	0.55**	0.50**			
%EMG	0.14	0.05	0.20	0.26	0.12	0.01	0.15		
EMG Rest	-0.17	-0.05	-0.27	-0.30	-0.25	0.03	-0.33	-0.72**	

*FIQ* Fibromyalgia Impact Questionnaire score, *PCS* Pain Catastrophizing Scale score, *STAI-A* State-Trait Anxiety Index State score, *STAI-B* State-Trait Anxiety Index Trait score, *PSS* Perceived Stress Scale score, *Pain* mean reported pain intensity across the protocol, *Stress *mean reported stress intensity across the protocol, *%EMG* mean normalized sEMG across the protocol. * = significant at *p* < 0.05 level; ** significant at *p* < 0.001 level

**Table 9:** Correlations of the scores in psychological questionnaires with psychophysiological measures within the control group (Relations between the psychological and psychophysiological measures)

	STAI-A	STAI-B	PSS	Pain	Stress	%EMG	EMG Rest
STAI-B	0.61**						
PSS	0.55**	0.76**					
Pain	0.12	0.24	0.12				
Stress	0.01	-0.04	0.06	-0.07			
%EMG	0.14	0.19	0.26	-0.09	0.19		
EMG Rest	-0.34	-0.31	-0.24	0.13	-0.25	-0.55**	

*STAI-A* State-Trait Anxiety Index State score, *STAI-B* State-Trait Anxiety Index Trait-score, *PSS *Perceived Stress Scale score, *Pain *mean reported pain intensity across the protocol, *Stress *mean reported stress intensity across the protocol, *%EMG *mean normalized sEMG across the protocol. * = significant at *p* < 0.05 level; ** significant at *p* < 0.001. level

In patients, pain correlated positively with perceived stress (*r* = 0.45, *p* < 0.001), concurrent anxiety (*r* = 0.38, *p* < 0.001), and trait-anxiety (*r* = 0.30, *p* < 0.05), but in controls correlations were less (between 0.12 and 0.24). Similarly, stress during the procedure correlated in patients with perceived stress (*r* = 0.55, *p* < 0.001), concurrent anxiety (*r* = 0.57, *p* < 0.001), and trait anxiety (*r* = 0.51, *p* < 0.001), but, again, less in controls (-0.07 to 0.06),.

Correlations between %EMG or EMG rest time variables and psychological questionnaires were not statistically significant.

Of the EMG variables there were only small positive correlations between %EMG and psychological measures (the greatest being 0.26 for trait anxiety in patients, and 0.26 for perceived stress in controls). Between resting EMG and psychological measures, there were small negative correlations (the greatest being − 0.30 for trait anxiety in patients, and − 0.34 for concurrent anxiety in controls).

There was a small negative correlation between resting EMG and stress during the procedure (*r* = -0.33 in patients, *r* = 0.25 in controls), otherwise correlations between psychophysiological measures, pain, and stress were less (-0.09 to 0.19).

## Discussion

### Muscle tension and muscular responses

The main findings of this study were that, as hypothesized, FM patients had higher %EMG and shorter EMG rest time values than the controls. Interestingly, cognitive stress did not cause significant changes in %EMG or EMG rest time in either group.

Unlike in the controls, %EMG decreased significantly in the FM patients during the final relaxation phase compared with the first phase. This may indicate higher anticipatory muscle tension in the FM patients than in the controls.

In a previous study with a similar study setting, Thieme et al. reported FM patients to have higher sEMG values than healthy controls, while we found the opposite. They measured the absolute sEMG of the trapezius muscles and reported values of the bilateral trapezius for the FM patients, with averages ranging from 7.03 to 8.35 µV during the protocol, similar to the values we measured. However, the values for their controls were much higher, ranging from 14.52 to 22.59 µV (in contrast to the mean of 4.87µV in our study). Thus, our conflicting findings may be explained by the differences in the control groups, although these are not obvious: the control groups were both all-female and similar in age (mean age 46 vs. 48 years) with no obvious demographic difference [8].

Within the FM patient subgroup, higher FIQ score predicted a longer EMG rest time. This was surprising, as we expected the patients with more symptoms to have a shorter EMG rest time. We speculate this is likely due to confounding factors. For example, in *post-hoc* testing, the patients with high FIQ scores and low leisure time physical activity tended to have shorter EMG rest times, while patients with high FIQ scores and who were also physically active tended to have EMG rest times similar to the patients with low FIQ scores, regardless of leisure time physical activity levels, though this was not statistically significant in the small subgroups.

Both %EMG and EMG rest time were calculated in relation to MVC, which was lower in FM patients than in controls. This contributes to the patients having higher %EMGs and shorter EMG rest times. The absolute sEMG voltages were also significantly higher in the left trapezius and right biceps of the FM patients, with a tendency for higher values in the other muscles. Thus, our results support the theory of increased resting muscle tension in FM. As discussed above, this is in contrast to previous work by Thieme et al., reporting lower muscle activity, and Westgaard et al., reporting similar muscle activity in FM patients compared with healthy controls [8, 9].

The MVCs of FM patients were lower than those of the controls in the upper extremities but comparable to controls in erector spinae muscles (in the trunk). This might indicate decreased neuromuscular control or upper body muscle mass of the patients. Westgaard et al. found the MVC of the FM patients for the trapezius, deltoid, and biceps brachii muscles to be 55 to 69 % of the MVC of the controls [9], results consistent with our study.

### Associations between the recorded physiological responses, pain, and psychological factors

Perceived stress during the stressful procedure seemed to be strongly linked to habitual experience of stress and anxiety in FM patients but not in healthy controls.

As anxiety is known to be connected to the severity of chronic pain, we expected anxiety and pain to be correlated. Indeed, overall pain during the protocol correlated with anxiety in the FM patients in both the linear modelling and the correlation coefficients.

However, neither %EMG nor EMG rest time correlate with any of the psychological measures in either subgroup. This suggests that short-term reactivity at least of muscle activity is not strongly linked to the psychological measures we used and may be mediated through other mechanisms.

The FM patients showed an increase in pain during the second stress phase. Consistent with our findings, Crettaz et al. reported that stress lowers the pressure pain threshold of FM patients [25]. Similar to the study by Thieme et al., our patients reported significantly higher stress levels than did controls across the entire test period [8].

FM patients often report an inability to relax. This phenomenon could be connected to the higher baseline values of sEMG and stress that we observed and is consistent with the observed reduced rest time. It is unclear to what degree lower physical activity is reflected in lowered MVC of the FM patients.

### Strengths and limitations of the study

Our sample size did not allow for controlling all possible confounding factors, which limits the generalizability of the results. However, the patient group was representative of the population encountered in clinical settings, with many comorbidities and medications. As a measure of FM severity, the FIQ score can be used to estimate the similarity of our patient population to those in previous studies. For example, compared with the validation study of the Finnish-language FIQ by Gauffin et al., the FM patients had similar mean age (45.1 vs. 47 years), BMI (28.2 vs. 28.1 kg/m^2^), and FIQ (44.5 vs. 49.8) [20].

A single researcher conducted all measurements and clinical examinations, which minimized interobserver variation but did not allow for interrater reliability checks.

The induction of stress in our study was successful, both groups reporting higher perceived stress during the phases of cognitive stress.

Our study was not designed to measure dynamic muscle function. Accordingly, the conclusion of dynamic, rather than static, muscle function disturbance is not definitive. We strongly encourage further studies along this line of inquiry.

## Conclusion

Our study revealed that repeated cognitive stress increases pain intensity in FM patients. The increase in pain did not seem to be directly linked to an increase in muscle tension, as we did not observe that acute cognitive stress affected this. In accordance with earlier studies, we found lower maximum muscle function and increased resting muscle tension in FM patients compared with healthy controls. The role of muscle activity in flare-ups remains unclear, but stress and anxiety are implicated as promising targets in treatment of FM.

## Data Availability

The datasets generated and analysed during the current study are not publicly available as consent for this was not asked from the study subjects. The data are available from the corresponding author on reasonable request if also approved by our ethics committee.
